# Effects of supplementing narasin to *Bos indicus* heifers during late-gestation and lactation on development of the offspring

**DOI:** 10.1093/tas/txad023

**Published:** 2023-02-28

**Authors:** Victor F B Miranda, Reinaldo F Cooke, Pedro V F Lasmar, Carlos E D Silva, Samir Burato, Caio L C Aguiar, Hingryd A O Ferreira, Eduardo A Colombo, Rodrigo L Valarelli, Tiago Leiva, Jose L M Vasconcelos

**Affiliations:** Faculdade de Medicina Veterinária e Zootecnia, Universidade Estadual Paulista, Botucatu, SP 18618-970, Brazil; Department of Animal Science, Texas A&M University, College Station, TX 77845, USA; Elanco Animal Health, Sao Paulo, SP 04703-002, Brazil; Faculdade de Medicina Veterinária e Zootecnia, Universidade Estadual Paulista, Botucatu, SP 18618-970, Brazil; Faculdade de Medicina Veterinária e Zootecnia, Universidade Estadual Paulista, Botucatu, SP 18618-970, Brazil; Faculdade de Medicina Veterinária e Zootecnia, Universidade Estadual Paulista, Botucatu, SP 18618-970, Brazil; Faculdade de Medicina Veterinária e Zootecnia, Universidade Estadual Paulista, Botucatu, SP 18618-970, Brazil; Department of Animal Science, Texas A&M University, College Station, TX 77845, USA; Elanco Animal Health, Sao Paulo, SP 04703-002, Brazil; Elanco Animal Health, Sao Paulo, SP 04703-002, Brazil; Faculdade de Medicina Veterinária e Zootecnia, Universidade Estadual Paulista, Botucatu, SP 18618-970, Brazil

**Keywords:** beef cows, gestation, narasin, offspring, production

## Abstract

This experiment evaluated the effects of supplementing narasin during late-gestation and lactation on productive and physiological responses of *Bos indicus* beef heifers and their offspring. Pregnant, nulliparous Nelore heifers (*N* = 88) that conceived under the same fixed-time artificial insemination protocol and to the same sire were used. Heifers were ranked by maternal ability genomic score, body weight (**BW**) and body condition score (**BCS**) and allocated to 44 drylot pens (2 heifers per pen; 10 × 25 m). Pens were ranked by these traits and alternatively assigned to receive (**NAR**) or not (**CON**) 0.260 mg of narasin/kg of heifer BW daily (Elanco Saúde Animal, São Paulo, Brazil). Narasin was mixed into a supplement offered at 0.30% of heifer BW from day 0 until heifers weaned their calves (day 316), whereas CON heifers received the same supplement without narasin addition. Heifers received *Urochloa brizantha* hay and water for ad libitum consumption (days 0 to 316) and calved between days 97 to 112 of the experiment. After calving, heifers and offspring had access to hay and supplement; hence, supplements and narasin were offered according to heifer + calf BW beginning on day 162. No treatment differences were detected (*P* ≥ 0.18) for heifer BW and BCS during the experiment, although BW loss from day 0 to calving was less (*P* = 0.04) in NAR compared to CON heifers. Hay intake during the experiment did not differ (*P* = 0.79) between treatments. Serum IGF-I concentrations were greater (*P* = 0.05) for NAR heifers on day 60 of the experiment and did not differ (*P* ≥ 0.28) between treatments 24 h and 30 d after calving (treatment × day interaction; *P* = 0.04). No treatment effects were detected (*P* ≥ 0.58) for calf birth BW. Serum concentrations of total protein 24 h after birth were greater (*P* = 0.04) in calves from NAR compared with CON heifers, and a tendency (*P* = 0.10) for a similar outcome was noted for serum IgG concentrations. Diarrhea incidence did not differ (*P* = 0.16) between treatments, although the number of total diarrhea cases per calf were greater (*P* = 0.03) in the CON offspring. Growth rate of calves from NAR heifers tended (*P* = 0.08) to be greater, resulting in heavier calves at weaning (*P* ≤ 0.04) compared with CON offspring. Collectively, these outcomes indicate narasin supplementation to beef heifers as a nutritional alternative to improve cow–calf productivity via developmental programming effects during gestation, as well as direct consumption by their nursing offspring.

## INTRODUCTION

Maternal nutrition is a major extrinsic factor programming nutrient partitioning and development of fetal organ systems associated with health, production, and reproduction (Funston et al., 2010). Nutritional management of late-gestating beef cows directly impacts performance of the offspring via developmental programming effects (Moriel et al., 2021). Bohnert et al. (2013) and Marques et al. (2016) reported that beef cows with body condition score (**BCS**) ≥ 5.0 during late gestation weaned heavier calves compared to cohorts with BCS < 5.0. In addition to postnatal growth, nutritional status of gestating cows can also impact offspring immunity (Moriel et al., 2020). Calves born from cows with restricted feed intake during late gestation had less serum IgG concentrations, even if provided colostrum with adequate IgG content, compared to calves born from cows in adequate nutritional status (Hough et al., 1990). Hence, management to ensure adequate nutrition for gestating beef cows is critical for cow–calf productive efficiency.

Ionophores are feed additives that modulate rumen function, improving energy efficiency of ruminal fermentation and energy supply to the host (Bergen e Bates, 1984). Narasin is an ionophore widely used for forage-fed *B. indicus* cattle, typical of cow–calf systems in Brazil (Marques and Cooke, 2021). Narasin supplementation favored propionate synthesis in the rumen (Soares et al., 2021), did not impact voluntary feed intake (Cappellozza et al., 2019), and improved cattle body weight (**BW**) gain (Limede et al., 2021). Vedovatto et al. (2022) recently reported that supplementing the ionophore monensin to Brangus multiparous beef cows during late gestation resulted in heavier calves at weaning. Ionophore supplementation may be of greater benefit to replacement heifers, particularly during first gestation and lactation when these young females have increased nutritional demands compared with mature cows (NASEM, 2016). Therefore, we hypothesized that supplementing narasin to beef heifers is a nutritional alternative to improve their offspring productivity. To test this hypothesis, this experiment evaluated the effects of narasin supplementation during late-gestation and lactation on productive and physiological responses of beef heifers and their offspring.

## MATERIALS AND METHODS

All animals utilized in this experiment were cared for in accordance with acceptable practices and experimental protocols reviewed and approved by the Universidade Estadual Paulista, Comitê de Ética no Uso de Animais (#0195/2019).

### Animal Management and Dietary Treatments

Eighty-eight nulliparous, pregnant Nelore heifers (BW = 364 ± 4 kg, BCS = 5.60 ± 0.04 according to Wagner et al., 1988) were assigned to the experiment at the end of their 2nd trimester of gestation (day 0 of the experiment; ~21 mo of age). All heifers were pregnant to the same fixed-time artificial insemination protocol using semen from a single Nelore bull, according to the breeding management and pregnancy diagnosis described by Rodrigues et al. (2014). At the beginning of the experiment (day 0), all heifers were at day 185 of pregnancy.

All heifers were evaluated for maternal ability (120 d) using the Clarifide Nelore genomic test (Zoetis Animal Health, São Paulo, Brazil) 30 d prior to the beginning of the experiment. On day −14, heifers were ranked by maternal ability (Table 1), BW, and BCS, and allocated to 44 drylot pens (2 heifers per pen; 10 × 25 m) in a manner that these traits were similar between heifers within the same pen. Pens were ranked by maternal ability, mean BCS, and mean BW, and alternatingly assigned to receive (**NAR**) or not (**CON**) 0.260 mg of narasin/kg of heifer BW daily (Elanco Saúde Animal). Narasin was mixed into a supplement (Table 2) offered at 0.30% of heifer BW from day 0 until heifers weaned their calves (day 316 of the experiment), whereas CON heifers received the same supplement without the inclusion of narasin. Heifers calved between days 97 and 112 of the experiment. Supplements were fed daily and consumed within 3 h after feeding across all pens. Throughout the experimental period (days 0 to 316), heifers received *Urochloa brizantha* hay and water for ad libitum consumption. Hay and supplement were offered separately in different feed bunks, whereas both dams an offspring had access to hay and supplement after calving. Narasin was administered (0.260 mg/kg of heifer BW) according to Cappellozza et al. (2019) and Limede et al. (2021). Supplement (and narasin to NAR heifers) was offered according to heifer + calf BW beginning on day 162 of the experiment to account for calf consumption. Heifer dams and calves were vaccinated against *Clostridium* (Poli-star, Vallée, Minas Gerais, Brazil) on day 245, and against foot-and-mouth disease (Vallée Aftosa, Vallée) on day 286. Calves were also revaccinated against *Clostridium* (Poli-star, Vallée) on day 286.

**Table 1.** Genomic potential of heifers (Clarifide Nelore, Zoetis Animal Health, São Paulo, Brazil) assigned to receive (**NAR**) or not (**CON**) 0.260 mg of narasin/kg of heifer BW daily (Elanco Saúde Animal)^1^

Item	CON	NAR	SEM	*P-*value
Calf body weight (120 d)	−1.52	−1.19	0.35	0.50
Calf body weight (210 d)	−1.68	−1.04	0.43	0.30
Maternal ability (120 d)	−0.601	−0.659	0.286	0.88
Maternal ability (210 d)	−0.278	−0.444	0.315	0.71
Calf *Longissimus* muscle area	−0.743	−0.390	0.308	0.22
Calf backfat thickness	−0.157	−0.123	0.041	0.56
Calf body structure (weaning)	42.2	43.7	1.7	0.56
Calf precocity (weaning)	43.3	45.6	1.5	0.29
Calf muscularity (weaning)	40.0	42.3	2.4	0.35
Calf height (weaning)	0.382	0.396	0.135	0.89

^1^Treatments were offered to heifers from day 0 of the experiment (day 185 of gestation) until day 316 of the experiment (weaning of the offspring). Heifers were evaluated 30 d prior to beginning of the experiment.

**Table 2.** Nutritional profile of hay and supplement offered to heifers during the experiment^1^

Item	Hay	Supplement
Dry matter, %	91.2	89.4
Total digestible nutrients, %	52.8	59.1
Neutral detergent fiber, %	74.1	15.1
Acid detergent fiber, %	46.3	9.2
Crude protein, %	4.0	32.0

^1^Based on wet chemistry procedures by a commercial laboratory (3R labs, Lavras, Minas Gerais, Brasil). Supplement included limestone, sodium chloride, sulfur, soybean meal, calcium monophosphate, ground corn, calcium iodate, manganese oxide, zinc oxide, sodium selenite, cobalt sulphate, copper sulfate, and urea. Formula is proprietary (Cargill Nutrição Animal, Goiânia, Goiás, Brazil).

### Sampling

Samples of hay and supplements were collected monthly, pooled across months, and analyzed for nutrient content by a commercial laboratory (3R labs, Lavras, Minas Gerais, Brasil). Samples were analyzed by wet chemistry procedures for concentrations of CP (method 984.13; AOAC, 2006), ADF (method 973.18 modified for use in an Ankom 200 fiber analyzer, Ankom Technology Corp., Fairport, NY; AOAC, 2006), and NDF (Van Soest et al., 1991; modified for Ankom 200 fiber analyzer, Ankom Technology Corp.).

Individual heifer unshrunk BW and BCS (Wagner et al., 1988) were recorded on days 0, 30, and 60 of the experiment. Heifers that failed to calve a live offspring or calved twins were removed from the experiment. Heifer unshrunk BW and BCS as well as calf unshrunk BW were recorded 24 h after calving, 30 d after calving [days 127 to 142 of the experiment (~day 134)], and then on days 162, 204, 245, 286, and 316 of the experiment. Calf growth rate was modeled by linear regression of BW against sampling days, and each regression co-efficient was used as individual growth response. Rate of supplementation and narasin delivery were adjusted according to these BW measurements. Heifers were also evaluated for intramuscular marbling, *Longissimus* muscle area, and backfat thickness via real-time ultrasonography on days 0, 60, 162, 204, 245, and 316 of the experiment (BIF, 2010; Paz et al., 2022).

Blood samples were collected from heifers on days 0 and 60 of the experiment, 24 h after calving, and 30 d after calving for analysis of serum insulin-like growth factor I (**IGF-I**; Cooke et al., 2012). Blood samples were collected from calves 24 h after calving for analysis of serum protein concentrations using a Brix refractometer (Deelen et al., 2014), as well as serum IgG concentrations (E11-118, Bethyl Laboratories, Montgomery, TX). All calves were observed to suckle their damns within 3 h after birth. Blood samples were collected via jugular venipuncture into commercial blood collection tubes (Vacutainer, 10 mL; Becton Dickinson, Franklin Lakes, NJ) containing no additive, placed immediately on ice, centrifuged (2,500 × *g* for 15 min) for serum harvest, and stored at −20 °C on the same day of collection. The intra- and inter-assay CV were, respectively, 2.1% and 5.2% for serum IGF-I and 3.5% and 4.1% for serum IgG.

Hay intake from each pen was evaluated in 5-d intervals by recording daily hay offer and collecting the nonconsumed hay daily (days –5 to 0 as baseline, days 25 to 30, days 55 to 60, days 90 to 95, days 120 to 125, days 157 to 162, days 199 to 204, days 240 to 245, days 281 to 286, and days 311 to 316). Samples of the nonconsumed hay from each pen were dried for 96 h at 50 °C in forced-air ovens for dry matter calculation. Hay intake of each pen was divided by the number of sampling days and heifers within each pen, and expressed as kg per heifer/d. Calf health was monitored daily (Dewell et al., 2006), whereas the only health disorder noted herein was diarrhea. Calves detected with diarrhea received enrofloxacin (Baytril 100, Elanco Saúde Animal) as initial treatment, and sulfadoxine + trimethoprim (Borgal, MSD Saúde Animal, São Paulo, Brazil) as secondary treatment. Calves were considered cured if no diarrhea symptoms were observed for three consecutive days, and a new diarrhea case diagnosed if symptoms were observed after this 3-d period (Dewell et al., 2006).

### Statistical Analysis

All data were analyzed with pen as the experimental unit, and pen (treatment) and heifer (pen) as random variables. Quantitative data were analyzed using the MIXED procedure of SAS (SAS Inst. Inc., Cary, NC), binary data were analyzed using the GLIMMIX procedure of SAS (SAS Inst. Inc.), and Satterthwaite approximation to determine the denominator df for tests of fixed effects. All model statements included the effects of treatment, in addition to day treatment × day interaction for repeated measures. Heifer carcass composition and serum IGF-I concentrations on day 0, as well as hay intake recorded from days −5 to 0 were used as covariates for each respective analysis. Model statements for calf-related responses also included calf gender as independent covariate. The specified term used in the repeated statements was day, the subject was heifer (pen), and the covariance structure used was autoregressive, which provided the best fit for these analyses according to the lowest Akaike information criterion. Results are reported as least square means, covariately adjusted when applicable (carcass composition, serum IGF-I, hay intake, and calf-related responses), and separated using least square differences. Significance was set at *P* ≤ 0.05, and tendencies were determined if *P* > 0.05 and ≤ 0.10.

## RESULTS AND DISCUSSION

A total of 23 heifers were excluded from the experiment (9 failed to calve, 7 calved twins, 2 for excitable temperament, and 5 due to injuries unrelated to treatments), and all their individual results obtained prior to removal were discarded. Accordingly, 40 pens (20 per treatment) remained until the completion of the experiment, which included pens with a single heifer and her offspring. Table 1 reports the genomic potential of heifers according to the Clarifide test, including maternal ability at 120 d. None of these traits differed (*P* ≥ 0.22) between treatments suggesting similar genetic potential of offspring from NAR and CON heifers, particularly because all calves were sired by the same bull.

**Figure 1.**
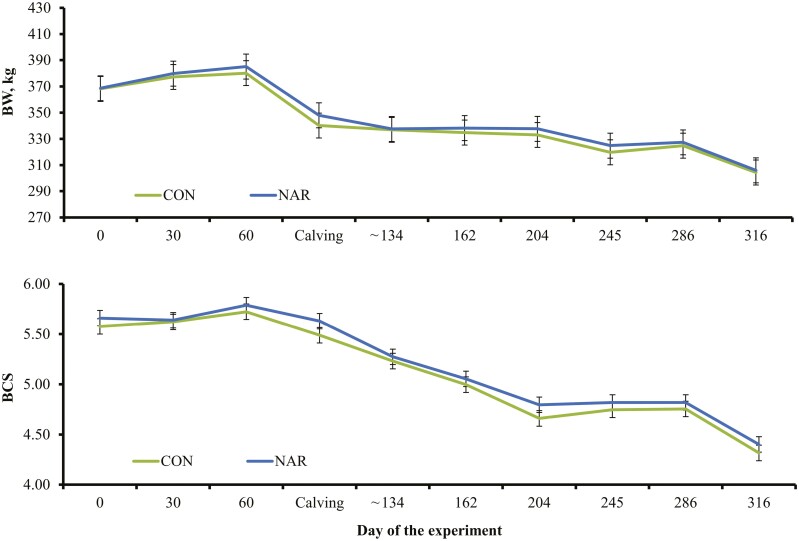
Body weight (**BW**) and body condition score (**BCS**; according to Wagner et al., 1988) of beef heifers supplemented (**NAR**) or not (**CON**) with 0.260 mg of narasin/kg of heifer BW daily (Elanco Saúde Animal). Treatments were offered to heifers from day 0 of the experiment (day 185 of gestation) until day 316 of the experiment (weaning of the offspring). Cows calved from days 97 to 112 of the experiment, and the subsequent BW and BCS recorded 30 d after calving (~day 134 of the experiment). No treatment differences were detected (*P* ≥ 0.33).

No treatment differences were detected (*P* ≥ 0.18) for heifer BW, BCS, and carcass composition during the experimental period (Table 3 and Figure 1). Loss in BW from the beginning of the experiment to 24 h postcalving was less (*P* = 0.04) in NAR heifers compared to CON heifers, which was not sufficient to impact BCS and BW values. These outcomes do not corroborate the established benefits of ionophores to cattle performance (Marques and Cooke, 2021), including narasin supplementation to Nelore cattle receiving a forage-based diet (Limede et al., 2021). Nonetheless, heifers from both treatments lost BW and BCS during the majority of the experimental period, which can be associated with the poor quality of the hay. Ionophores may become ineffective in cattle consuming diets based on low-quality forages due to limited feed intake and availability of nonstructural carbohydrates, decreasing the precursors for ruminal propionate production (Rowe et al., 1991). Vendramini et al. (2015) reported that monensin supplementation did not improve growth rates of beef heifers grazing low-quality pastures and supplemented with concentrate at 0.1% of their BW. Hence, narasin supplementation likely failed to substantially improve heifer growth responses herein due limited availability of nutrients from the basal diet.

**Table 3.** Performance responses of beef heifers supplemented (**NAR**) or not (**CON**) with 0.260 mg of narasin/kg of heifer BW daily (Elanco Saúde Animal)^1^

Item	CON	NAR	SEM	*P-*value
Heifer BW^2^				
Initial (day 0)	367	369	10	0.77
Calving (24 h later)	339	348	9	0.28
BW change	−28	−20	2	0.04
Weaning (day 316)	303	304	9	0.92
BW change	−36	−42	5	0.37
BCS^2^				
Initial (day 0)	5.56	5.65	0.07	0.38
Calving (24 h later)	5.46	5.59	0.10	0.18
BW change	−0.08	−0.02	0.07	0.59
Weaning (day 316)	4.31	4.39	0.08	0.46
BW change	−1.14	−1.19	0.10	0.60
Hay intake,^3^ kg/d (dry matter)	9.25	9.30	0.15	0.79
Carcass composition^4^				
Backfat thickness, mm	2.82	2.80	0.28	0.91
Longissimus muscle area, cm^2^	48.7	49.2	1.2	0.76
Marbling score	3.57	3.52	0.17	0.77

^1^Treatments were offered to heifers from day 0 of the experiment (day 185 of gestation) until day 316 of the experiment (weaning of the offspring).

^2^Body weight (**BW**) was unshrunk and body condition score (**BCS**) according to Wagner et al. (1988).

^3^Evaluated in 5-d intervals by recording daily hay offer and collecting the nonconsumed hay daily (days −5 to 0 as baseline days 25 to 30, days 55 to 60, days 90 to 95, days 120 to 125, days 157 to 162, days 199 to 204, days 240 to 245, days 281 to 286, and days 311 to 316). Values from days −5 to 0 were used as independent covariate.

^4^Evaluated via real-time ultrasonography on days 0, 60, 162, 204, 245, and 316 of the experiment (BIF, 2010; Paz et al., 2022). Values from day 0 were used as independent covariate.

**Figure 2.**
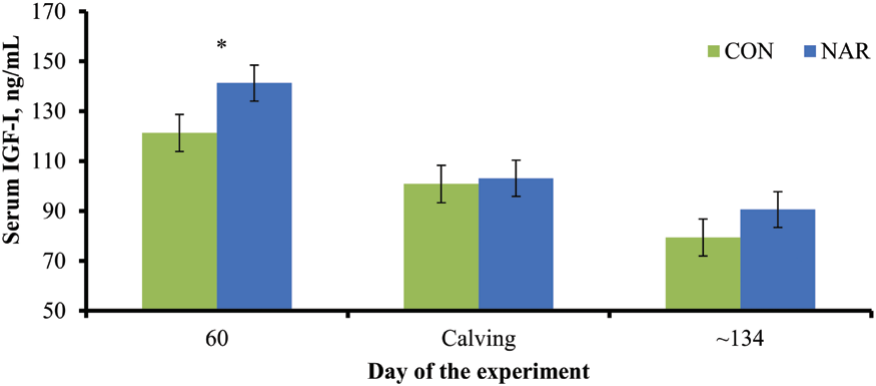
Serum concentrations of insulin-like growth factor I (**IGF-I)** in beef heifers supplemented (**NAR**) or not (**CON**) with 0.260 mg of narasin/kg of heifer BW daily (Elanco Saúde Animal). Treatments were offered to heifers from day 0 of the experiment (day 185 of gestation) until day 316 of the experiment (weaning of the offspring). Cows calved from days 97 to 112 of the experiment. Blood samples were collected from heifers on days 0 and 60 of the experiment, 24 h after calving, and 30 d after calving (~day 134 of the experiment). Results from day 0 were used as independent covariate. A treatment × day interaction was detected (*P* = 0.04), where ^*^P = 0.05.

Intake of hay also did not differ (*P* = 0.79) between treatments throughout the experiment (Table 3), indicating that narasin supplementation does not affect voluntary forage intake (Cappellozza et al., 2019; Marques and Cooke, 2021). Treatments also did not impact (*P* ≥ 0.70) hay intake prior to calving (7.97 vs. 7.95 kg of dry matter per heifer daily for CON and NAR, respectively; SEM = 0.15) nor during lactation (9.82 vs. 9.90 kg of dry matter per heifer daily for CON and NAR, respectively; SEM = 0.18) when calves also had access to hay. Moreover, hay intake did not differ (*P* ≥ 0.49) between treatments prior to calving as % of heifer BW (2.13 vs. 2.07% for CON and NAR, respectively; SEM = 0.10), and during lactation as % of heifer + calf BW (2.41% vs. 2.38% for CON and NAR, respectively; SEM = 0.05). In turn, a treatment × day interaction was detected (*P* = 0.04) for serum IGF-I concentrations (Figure 2), which was greater (*P* = 0.05) for NAR heifers on day 60 of the experiment and did not differ (*P* ≥ 0.28) between treatments 24 h and 30 d after calving. Although circulating IGF-I concentrations are often associated with feed intake, this hormone is considered a metabolic marker of nutritional status in cattle (Hess et al., 2005). Vendramini et al. (2018) reported increased circulating IGF-I concentrations in growing cattle consuming low-quality forages and supplemented with monensin. Perhaps narasin supplementation was effective in improving, at least marginally, heifer nutritional status during gestation when BW and BCS were increasing (Figure 1). In turn, narasin supplementation was ineffective in eliciting similar responses during lactation, when the basal diet failed to provide adequate nutrients for heifer BW and BCS maintenance or gain.

Despite exclusion of heifers due to reasons not pertaining to the objectives of this experiment, the % of heifers that calved a single live calf did not differ (*P* = 0.82) between treatments (Table 3). No treatment effects were detected (*P* ≥ 0.58) for calf birth BW (adjusted or not; BIF, 2010), as well as % of male calves born (Table 4). Other studies also reported that nutritional status of beef cows during gestation had no impact of calf birth BW (Stalker et al., 2006; Marques et al., 2016), including in late-gestating cows receiving or not ionophore supplementation (Vedovatto et al., 2022). However, serum concentrations of total protein 24 h after birth were greater (*P* = 0.04) in calves from NAR heifers compared with CON, and a tendency (*P* = 0.10) for a similar outcome was noted for serum IgG concentrations (Table 4). Deelen et al. (2014) reported that concentrations of total protein in serum measured via Brix refractometer is strongly and positively correlated with serum IgG concentrations. Circulating IgG in newborn calves is derived from the colostrum, which in turn is originated from maternal systemic synthesis and circulatory reserves of IgG (Hurley and Theil, 2011). Perhaps the increased serum IgG concentrations in calves from NAR heifers is resultant from heightened humoral immunity of their dams during late gestation (Brandão et al., 2020; Moriel et al., 2021). Calves from NAR heifers may also have had greater capacity to absorb IgG from the colostrum as consequence of improved developmental programming (Hough et al., 1990; Palmer et al., 2022). Vedovatto et al. (2022) reported that monensin supplementation to late-gestating beef cows increased serum IgG concentrations in their newborn calves. As noted by these latter authors, research investigating how nutritional management of gestating cows impacts colostrum quality and passive immune transfer is limited, and deserves investigation given its importance to life-long offspring development (Besser and Gay, 1994; Wittum and Perino, 1995).

**Table 4.** Calving and weaning responses from beef heifers and their offspring supplemented (**NAR**) or not (**CON**) with 0.260 mg of narasin/kg of heifer BW daily (Elanco Saúde Animal)^1^

Item	CON	NAR	SEM	*P-*value
Calving results				
Heifers that calved a single live calf, %	72.7	75.0	7.1	0.82
% of male calves born	52.9	60.5	9.7	0.58
Calf birth BW, kg	27.8	28.3	0.8	0.65
Adjusted calf birth BW,^2^ kg	30.1	30.6	0.8	0.65
Serum protein,^3^ mg/dL	7.93	8.41	0.17	0.04
Serum IgG,^3^ mg/mL	204	240	15	0.10
Weaning results				
Calf weaning age, d	205	206	2	0.39
Calf weaning BW, kg	134	142	3	0.04
Growth rate from birth to weaning, kg/d	0.514	0.553	0.017	0.08
Calf 205-d adjusted weaning BW,^2^ kg	134	143	3	0.02
Calves with diarrhea from birth to weaning, %	62.0	44.4	8.7	0.16
N of diarrhea cases per calf weaned	0.93	0.50	0.13	0.03

^1^Treatments were offered to heifers from day 0 of the experiment (day 185 of gestation) until day 316 of the experiment (weaning of the offspring). Cows calved from days 97 to 112 of the experiment. Narasin was offered according to heifer + calf BW beginning on day 162 of the experiment.

^2^ According to BIF (2010).

^3^ Blood samples were collected from calves 24 h after birth.

**Figure 3.**
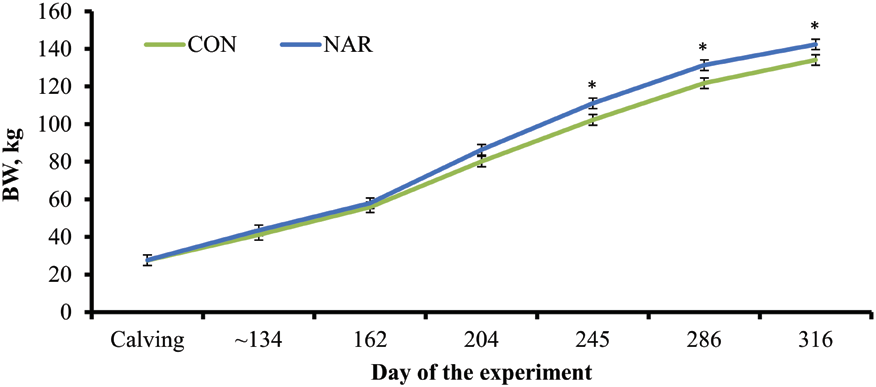
Body weight (**BW**) of calves from beef heifers supplemented (**NAR**) or not (**CON**) with 0.260 mg of narasin/kg of heifer BW daily (Elanco Saúde Animal, São Paulo, Brazil). Treatments were offered to heifers from day 0 of the experiment (day 185 of gestation) until day 316 of the experiment (weaning of the offspring). Cows calved from days 97 to 112 of the experiment, and the subsequent calf BW recorded 30 d after calving (~day 134 of the experiment). Narasin was offered according to heifer + calf BW beginning on day 162. A tendency for a treatment × day interaction was detected (*P* = 0.09), where ^*^P = 0.05.

No incidence of heifer or calf mortality was observed from birth to weaning. As expected, based on the experimental design, calf age at weaning did not differ (*P* = 0.39) between treatments (Table 4). Incidence of diarrhea also did not differ (*P* = 0.16) between treatments, although the number of total diarrhea cases per calf were greater (*P* = 0.03) in the offspring from CON heifers (Table 4). Growth rate of calves from NAR heifers tended (*P* = 0.08) to be greater, resulting in heavier calves at weaning (*P* ≤ 0.04) compared with calves from CON heifers (Table 4; Figure 3). As previously noted, calves had access to treatments until weaning and supplementation rate was adjusted at 0.3% of heifer + calf BW beginning on day 162. Hence, increased growth performance noted in NAR calves is likely resultant from a combination of factors, including: 1) a potential improvement in the nutritional status of NAR heifers during gestation, based on serum IGF-I concentrations prior to calving, which yielded developmental programming effects that promoted offspring postnatal growth (Vedovatto et al., 2022); 2) heightened postnatal immunity of NAR calves due to increased serum IgG concentrations after birth (Wittum and Perino, 1995), which reduced total number of diarrhea cases and benefited calf growth (Anderson et al., 2003); 3) consumption of narasin by NAR calves directly improved their health and growth performance, given that nutrient intake of calves (milk + hay + supplement) was adequate to support calf growth and yield the expected benefits from narasin supplementation (Cappellozza et al., 2019; Limede et al., 2021).

In conclusion, supplementing narasin to *B. indicus* beef heifers during late-gestation yielded marginal improvements in their nutritional status, such as increasing serum IGF-I concentrations and reducing BW loss until calving. Narasin supplementation to these heifers after calving did not improve their BW and BCS, likely due to limited dietary quality along with heightened nutritional demands from lactation. Nonetheless, supplemental narasin improved postnatal offspring health and growth responses. These outcomes can be associated with potential developmental programming effects, improved passive immunity, and direct narasin consumption by calves. Hence, narasin supplementation appears to be a nutritional alternative to improve cow–calf productivity, particularly in systems based on low-quality forages.
